# Spectroscopic detection of cotton Verticillium wilt by spectral feature selection and machine learning methods

**DOI:** 10.3389/fpls.2025.1519001

**Published:** 2025-05-15

**Authors:** Weinan Li, Lisen Liu, Jianing Li, Weiguang Yang, Yang Guo, Longyu Huang, Zhaoen Yang, Jun Peng, Xiuliang Jin, Yubin Lan

**Affiliations:** ^1^ College of Electronic Engineering (College of Artificial Intelligence), South China Agricultural University, Guangzhou, Guangdong, China; ^2^ National Center for International Collaboration on Precision Agricultural Aviation Pesticide Spraying Technology, South China Agricultural University, Guangzhou, Guangdong, China; ^3^ Nanfan Research Institute, Chinese Academy of Agricultural Sciences, Sanya, Hainan, China; ^4^ Cotton Research Institute, Chinese Academy of Agricultural Sciences, Anyang, Henan, China; ^5^ Institute of Crop Sciences, Chinese Academy of Agricultural Sciences, Beijing, China

**Keywords:** *Gossypium hirsutum*, *Verticillium dahliae*, spectral feature, feature selection, disease detection

## Abstract

**Introduction:**

Verticillium wilt is a severe soil-borne disease that affects cotton growth and yield. Traditional monitoring methods, which rely on manual investigation, are inefficient and impractical for large-scale applications. This study introduces a novel approach combining machine learning with feature selection to identify sensitive spectral features for accurate and efficient detection of cotton Verticillium wilt.

**Methods:**

We conducted comprehensive hyperspectral measurements using handheld devices (350–2500 nm) to analyze cotton leaves in a controlled greenhouse environment and employed Unmanned Aerial Vehicle (UAV) hyperspectral imaging (400–995 nm) to capture canopy-level data in field conditions. The hyperspectral data were pre-processed to extract wavelet coefficients and spectral indices (SIs), enabling the derivation of disease-specific spectral features (DSSFs) through advanced feature selection techniques. Using these DSSFs, we developed detection models to assess both the incidence and severity of leaf damage by Verticillium wilt at the leaf scale and the incidence at the canopy scale. Initial analysis identified critical spectral reflectance bands, wavelet coefficients, and SIs that exhibited dynamic responses as the disease progressed.

**Results:**

Model validation demonstrated that the incidence detection models at the leaf scale achieved a peak classification accuracy of 85.83%, which is about 10% higher than traditional methods without feature selection. The severity detection models showed improved precision as disease severity of damage increased, with accuracy ranging from 46.82% to 93.10%. At the canopy scale, UAV-based hyperspectral data achieved a remarkable classification accuracy of 93.0% for disease incidence detection.

**Discussion:**

This study highlights the significant impact of feature selection on enhancing the performance of hyperspectral-based remote sensing models for cotton wilt monitoring. It also explores the transferability of sensitive spectral features across different scales, laying the groundwork for future large-scale early warning systems and monitoring cotton Verticillium wilt.

## 1 Introduction

Cotton, a pivotal agricultural commodity, is globally recognized for its essential contribution to the textile industry. However, annual cotton production is significantly threatened by various pathogens, among which *Verticillium dahliae* (*V. dahliae*) is particularly devastating, causing yield reductions of up to 30% (Zhu et al., 2021). The pathogenic progression of *V. dahliae* often remains latent until the advanced stages of infection, by which point substantial crop damage has already occurred, leading to severe economic losses (Calderon et al., 2015; Zhang et al., 2020). Current management strategies for cotton Verticillium wilt face significant challenges, primarily due to the delayed onset of visible symptoms, which complicates early intervention efforts (Wang et al., 2016). Traditionally, the detection of cotton Verticillium wilt has relied on phenotypic assessments conducted by trained agronomists. While practical for later stages of disease progression, this method is labor-intensive and limited by the latency of symptom expression (Bardak et al., 2021; Huang et al., 2023). As a result, there is an urgent need for innovative detection methodologies capable of identifying Verticillium wilt at early stages to mitigate its impact effectively. Additionally, precision in traditional indoor germplasm screening is critical to ensure the selection of disease-resistant cultivars, thereby enhancing agricultural productivity and profitability.

Remote sensing has emerged as a promising technique for the non-destructive detection of crop diseases across multiple spatial scales (Liu et al., 2024; Wang et al., 2024; Zhu et al., 2024). Sensitive spectral indicators can detect subtle changes in physiological parameters or light protection mechanisms in plants under biological stress, providing early signals of disease (Mahlein et al., 2018; Morel et al., 2018; Zarco-Tejada et al., 2018). This spectral technique is increasingly being applied to the early detection of cotton Verticillium wilt and the examination of leaf biochemical parameters (Calderon et al., 2015; Shin et al., 2023; Yang et al., 2024). Radiative Transfer Models (RTMs) offer advantages in interpreting and modeling spectral changes caused by pathogen infections, making them valuable for plant disease detection at various scales (Morel et al., 2018; Hornero et al., 2020). Additionally, Continuous Wavelet Transform (CWT) has proven effective in capturing subtle spectral signals induced by pathogen infections (Shi et al., 2018a; Zhao et al., 2022). Spectral indices (SIs) derived from specific bands in CWT can be highly sensitive to the spectral responses caused by target plant diseases (Mahlein et al., 2010; Zhang et al., 2019).

Recent studies have demonstrated that combining feature subsets can significantly improve the efficiency of classification models without compromising accuracy (Huang et al., 2019; Hamed et al., 2020; Tian et al., 2021). In particular, machine learning classifiers integrated with feature selection algorithms have been successfully applied to reflectance spectra for the early detection of crop diseases, enhancing both classification efficiency and performance (Tian et al., 2021; Yang et al., 2022, 2024). Narrow wavebands in hyperspectral data exhibit high sensitivity to subtle plant changes induced by diseases, enabling the differentiation of various disease types and the early detection of asymptomatic infections (Bai and Jin, 2024). These advancements highlight the potential of remote sensing and machine learning for revolutionizing the early detection and management of cotton Verticillium wilt, ultimately contributing to sustainable agricultural practices.

In recent years, there has been a growing body of research focused on monitoring cotton Verticillium wilt, aiming to understand how narrow wavebands affect the detection performance of the disease at different infection stages. Proximal remote sensing using handheld spectrometers has been widely implemented at the leaf scale (Jing et al., 2009; Chen et al., 2012, 2014), demonstrating the potential of sensitive spectral indicators for the early detection of Verticillium wilt (Yang et al., 2022, 2024). Additionally, studies have measured and analyzed physiological and biochemical parameters, such as pigments and photosynthetic parameters, under Verticillium wilt stress to elucidate the mechanisms underlying the spectral response to infection (Chen et al., 2010, 2018; Yuan et al., 2023). At the canopy scale, UAV remote sensing has shown significant advantages in assessing the severity of cotton Verticillium wilt. Multi-source feature fusion, combining visible images and spectral data, has proven effective in improving the accuracy of disease severity estimation (Kang et al., 2023; Ma et al., 2024; Li X. et al., 2024). Furthermore, the integration of UAV remote sensing with agricultural drones has provided a practical framework for precision fungicide application to manage cotton Verticillium wilt (Li W. et al., 2024). On a broader scale, satellite remote sensing using multispectral sensors has enabled the monitoring of the spatial and temporal distribution of cotton Verticillium wilt across large regions (Chen et al., 2011; Wang et al., 2015; Gui et al., 2024).

Despite these advancements, current research on cotton Verticillium wilt detection using hyperspectral data has several limitations. Recent studies (Jing et al., 2009; Chen et al., 2012) have predominantly relied on a single type of spectral feature, and the feature selection methods employed have been relatively uniform, potentially lacking adaptability to different types of spectral features. Moreover, while recent research (Yang et al., 2024) has focused on the incidence detection of cotton Verticillium wilt, there has been limited exploration of severity detection of leaf damage using feature selection and machine learning methods. To address these gaps, the overall goal of this study is to conduct spectral detection on cotton leaves infected with Verticillium wilt at different stages and to evaluate the feasibility of using Machine Learning models coupled with wrapper-based Feature Selection algorithms (ML-FS) to identify sensitive spectral features and perform disease detection.

The specific objectives are: (1) to investigate the physiological basis of leaf biochemical parameters in response to cotton Verticillium wilt infection and their relationship with spectral detection, (2) to identify spectral features, including spectral reflectance, wavelet coefficients, and SIs, using filter-based feature selection methods, and to determine DSSF between healthy and infected leaves, (3) to evaluate the classification accuracy and identify the Optimal Feature Combination (OFC) using the ML-FS algorithm at the asymptomatic, early, and mild infection stages and (4) to assess the severity of cotton leaf damage by Verticillium wilt at different severity grades of damage, providing a comprehensive understanding of disease progression and detection capabilities.

## 2 Materials and methods

### 2.1 Experimental design

**Figure 1:**
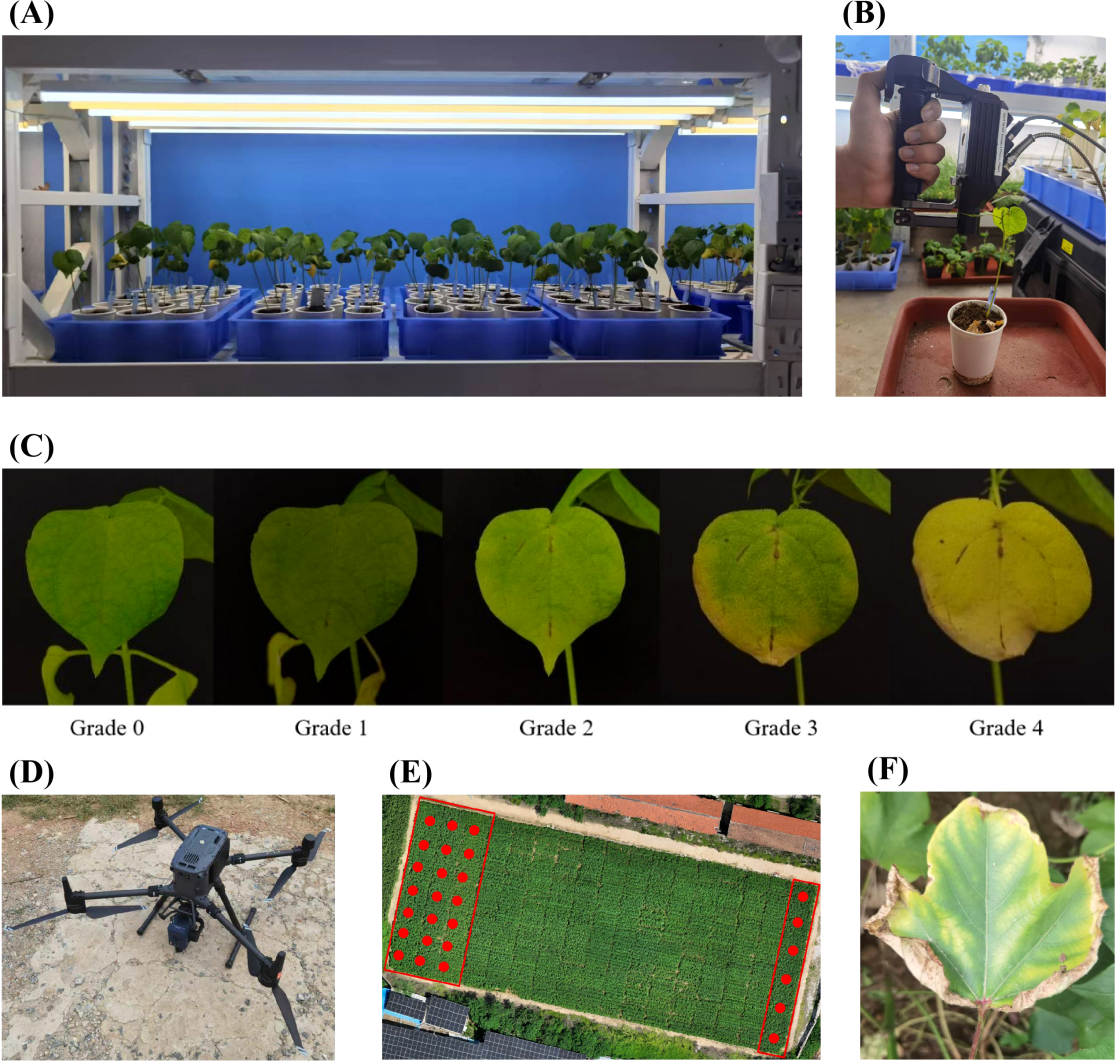
Diagram of experimental design and data collection. **(A)** The growth environment and planting method of cotton in Experiment 1 and Experiment 2. **(B)** Schematic diagram of non-imaging spectra acquisition for cotton leaves. **(C)** Images of cotton leaf at different severity grades of damage. **(D)** Schematic diagram of hyperspectral imaging for cotton canopy. **(E)** The growth environment and planting methods of cotton in Experiment 3. **(F)** Typical symptoms of cotton wilt disease in a field environment.

In the greenhouse environment, two cotton varieties—a moderately resistant variety, Baimian 1, and a susceptible variety, Zhongmiansuo 24—were cultivated under controlled conditions (**Figure 1A**). Plants of these varieties were divided into in two separate groups of 60 pots each, designated as **Experiment 1** and **Experiment 2**, to facilitate systematic data collection. Both experiments included those two varieties to uncover the universal spectral response patterns of cotton to Verticillium wilt, applicable to both susceptible and mid-resistant varieties. All plants were cultivated in a greenhouse environment, with a controlled photoperiod of 14 hours of light followed by 10 hours of darkness and a constant temperature of 25°C. After the first two true leaves had unfolded, all plants were inoculated with *V. dahliae*. For the *V. dahliae* treatments, 10 mL of conidial suspensions (10^7^ conidia/mL in sterile distilled water) of *V. dahliae* strain 991 was applied to the bottom of pots containing seedlings (Gong et al., 2017). Control plants were inoculated with an equal volume of sterile distilled water. For each plant, the first two true leaves were selected and marked, resulting in 120 samples for each experiment.

Data collection for Experiment 1 spanned the asymptomatic stage to the mid-infection phase, while for Experiment 2, it covered the early to late infection stages. Spectral measurements were conducted on the marked leaves at regular intervals across different Days After Inoculation (DAIs). Specifically, day collection occurred from DAI 10 to DAI 36 in Experiment 1 and from DAI 20 to DAI 40 in Experiment 2. Infected leaves were identified in 49 out of 120 samples in Experiment 1 and 76 out of 120 samples in Experiment 2. Comprehensive spectral data for both infected and healthy leaves were systematically recorded at each sampling interval.

A field environment (**Experiment 3**) was conducted in a cotton disease nursery (114°49′13.782″E, 36°06′47.412″N) located in Anyang County, Anyang City, Henan Province, China (**Figure 1E**). Two moderate resistant varieties, Baimian 1 and Zhongzhimian 2, and two susceptible varieties, Zhongmiansuo 24 and Jimian 11, were planted on April 26, 2024, with 50 plots allocated for each variety, totaling 200 plots. This setup allowed for the evaluation of spectral responses under natural field conditions, complementing the controlled greenhouse experiments. The cotton leaves in the experimental region showed significant symptoms of Verticillium wilt. (**Figure 1F**)

### 2.2 Data acquisition

Data collected in Experiment 1 and Experiment 2 included leaf hyperspectral reflectance and disease occurrence. Biochemical parameters—chlorophyll, carotenoids, anthocyanins, and water content—were estimated from reflectance spectra using the PROCWT model (Li et al., 2018). A FieldSpec 4 Hi-Res spectroradiometer (Analytical Spectral Devices, Boulder, USA) equipped with a leaf clip was used to measure leaf reflectance (**Figure 1B**). The spectral sampling interval was 1 nm across the 350–2500 nm range. A 1.5 m contact fiber-optic cable with a 25°field of view captured reflected light from the target. In both experiments, two fully unfolded true leaves per plant were marked, and spectral reflectance was measured at three points per leaf using the spectrometer’s leaf clip. The leaf clip, equipped with an active light source, ensured stable operation and minimal measurement errors in a controlled environment. Non-imaging spectral data were obtained by measuring each leaf 10 times near the center of the leaf vein, with the average value recorded as the spectral measurement. Spectral acquisition began at inoculation and continued at multiple time points post-inoculation. In Experiment 3, canopy-scale hyperspectral data in the field environment were collected using a UAV equipped with a hyperspectral camera (**Figure 1D**), consistent with previous research (Li W. et al., 2024).

Leaf classification was determined through visual interpretation by experts. After spectral measurements, leaves were categorized as infected or healthy based on the presence of Verticillium wilt symptoms. Leaves with stable disease spots on the final survey day were classified as infected, while those without spots were classified as healthy (Wang et al., 2004; Zhang et al., 2012). Disease severity of damage was graded as follows: **Grade 0**: No disease spots. **Grade 1**: Initial disease spots. **Grade 2**: Increased spot area with yellowing around leaf veins. **Grade 3**: Half-leaf symptomatic. **Grade 4**: Entire leaf yellowed or wilted (**Figure 1C**). A 5-level grading system was applied to all cotton plants in the study area, and the Disease Index (DI) was calculated for each plot (Zhang et al., 2023). Visual interpretation was performed by the same expert to ensure consistency across all experiments.

### 2.3 Biochemical parameters inversion

#### 2.3.1 Radiative transfer model inversion

The spectral specificity of cotton Verticillium wilt reflects the changes in physiological and biochemical parameters under infection. This study employed a radiative transfer model to invert hyperspectral signals and extract biochemical parameters, including chlorophyll (CHL), carotenoids (CAR), anthocyanins (ANT), and water content (EWT), at the leaf scale under Verticillium wilt infection.

The PROSPECT model (Jacquemoud and Baret, 1990) simulates leaf optical properties (reflectance and transmittance) in the optical domain from 400 nm to 2500 nm based on biophysical properties. Derived from the extended plate model (Allen et al., 1970), PROSPECT represents leaf optical properties as conical-hemispherical reflectance and transmittance, typically measured using an integrating sphere. These properties are often described as directional-hemispherical reflectance and transmittance, although such directional quantities are conceptual.

However, leaf reflectance measured with a leaf clip does not correspond to conical-hemispherical reflectance. As a result, comparing such reflectance with PROSPECT simulations (in forward or inverse mode) may lead to biased or uncertain results. To address this limitation, Li et al. (2018) developed the PROCWT method, which integrates the PROSPECT-D model (Féret et al., 2017) with CWT. PROCWT improves the retrieval of leaf biochemical parameters by reducing specular reflection effects and enhancing the absorption features of chemical constituents (Tian et al., 2021). The PROSPECT package in R (available at https://gitlab.com/jbferet/prospect/) was used to perform biochemical parameter inversion on spectral reflectance data and calculate all spectral indices.

#### 2.3.2 Statistical analysis methods

After the radiative transfer model inversion, significant differences in biochemical parameters between healthy and infected samples at different DAI were tested. Before conducting significant tests, all outliers were excluded. Outliers were defined as data points outside the three-fold standard deviation interval centered on the median and were excluded from statistical analysis (Yang et al., 2024).

Following outlier removal, normality tests were performed on the retained data for healthy and infected samples. For data conforming to a normal distribution, an independent t-test was applied (null hypothesis H₀: Infected = Healthy; alternative hypothesis H_a_: Infected ≠ Healthy; P< 0.05). For non-normally distributed data, the Mann-Whitney U-test was used.

The performance of ML-FS methodology was evaluated based on classification accuracy for separating infected and healthy samples at various infection stages. All statistical analyses, including normality tests and t-tests, were conducted using the Scipy Python library (available at https://github.com/scipy/scipy).

### 2.4 Detection of cotton leaf Verticillium wilt

**Figure 2:**
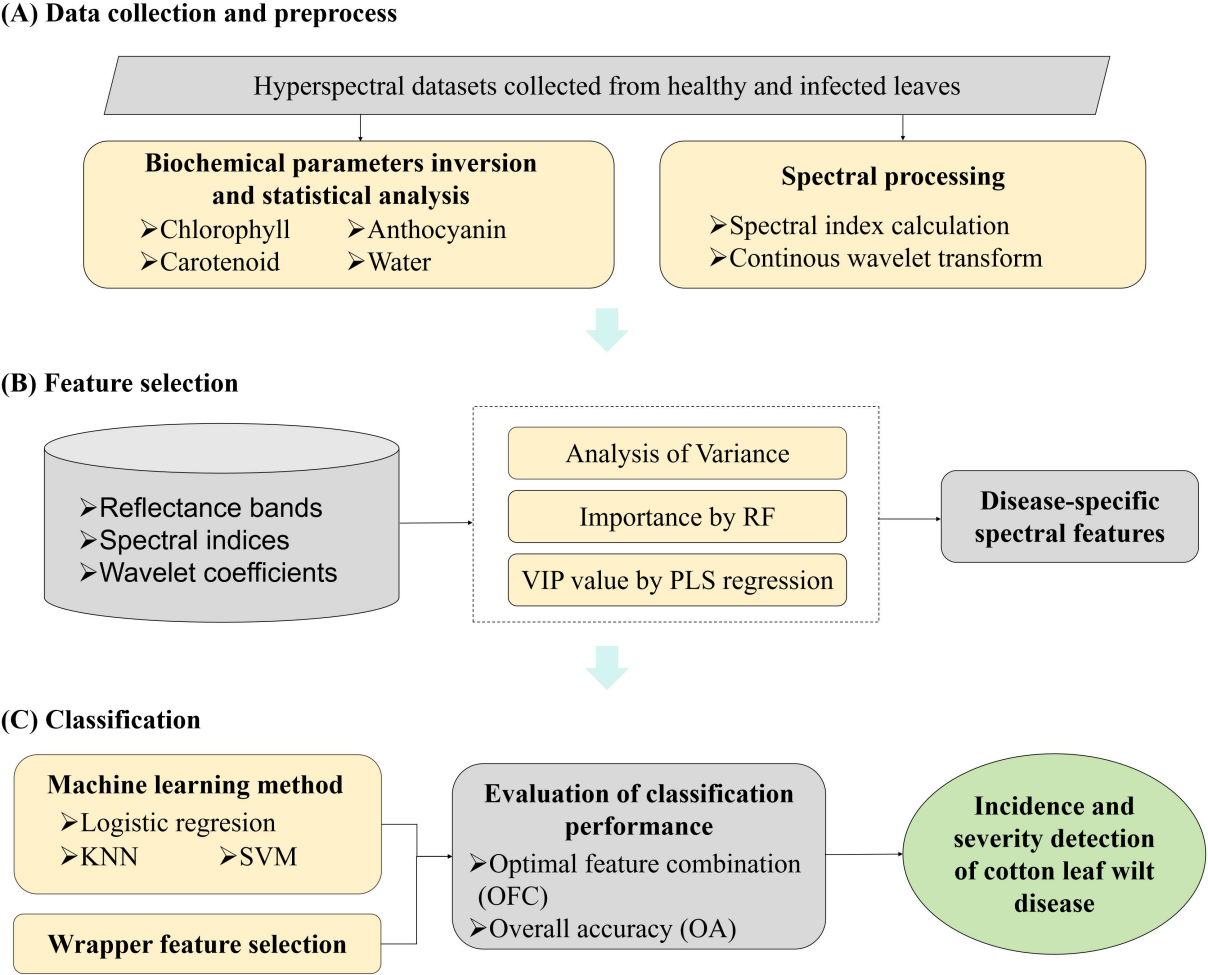
Schematic illustration of ML-FS classification methodology for the spectroscopic detection of cotton Verticillium wilt.

The detection of Verticillium wilt infection at different stages involved three main steps: data collection and preprocessing, feature selection, and classification (**Figure 2**). Details of data collection and preprocessing were described in the previous section. The radiative transfer model was used to invert biochemical parameters, while vegetation indices and wavelet coefficients were extracted from spectral reflectance data. In this section, targeted feature selection was performed using Analysis of Variance (ANOVA), Random Forest (RF) (Long et al., 2025), and Partial Least Squares (PLS) regression (Li et al., 2022) for different types of spectral features. Based on the selected DSSFs for cotton wilt disease, machine learning models—logistic regression (LR), K-Nearest Neighbor (KNN), and Support Vector Machine (SVM) (Yang et al., 2024)—were constructed to detect Verticillium wilt at the leaf scale. The transferability of these DSSFs across different scales and conditions was also investigated.

#### 2.4.1 Spectral feature extraction

Spectral features were extracted from the collected hyperspectral data of cotton leaves, resulting in three types of candidate features for Verticillium wilt detection: spectral reflectance, wavelet coefficients, and spectral indices. This refers to the reflectance of individual bands (1 nm width, 350–2500 nm) measured using a spectroradiometer. It captures changes in the amplitude of the reflection spectrum of cotton leaves following Verticillium wilt infection.

The observed spectral reflectance, a continuously changing signal, was processed using CWT. CWT separates changes in spectral reflectance at different frequencies by applying wavelet functions at various transformation scales. Compared to single-band reflectance, wavelet coefficients enhance weak spectral absorption characteristics, making them more sensitive to subtle changes. This study employed the Mexican hat wavelet function at transformation scales 2, 4, 6, 8, 10, 12, 14, and 16 (scales 1 to 8) (Yang et al., 2024). Wavelet coefficients were generated using the pywt Python library package (available at https://github.com/PyWavelets/pywt). Based on existing research, a total of 92 spectral indices across five categories were calculated as candidate features for detecting Verticillium wilt (**Supplementary Table 1**).

#### 2.4.2 Sensitive spectral feature selection for cotton Verticillium wilt

The number of spectral features extracted from the three categories (spectral reflectance, wavelet coefficients, and spectral indices) was extensive. Given the continuous nature of spectral reflectance and wavelet coefficients across the observation range, there was a significant correlation between adjacent bands. To enhance classification performance, improve learning accuracy, reduce computational costs, and increase model interpretability, feature selection methods were employed to reduce the dimensionality of the spectral feature dataset.

Reflectance features were evaluated using ANOVA due to their continuous nature. Spectral reflectance bands were ranked based on ANOVA scores, and redundant, irrelevant, or noisy features were removed. Wavelet coefficients, derived from reflectance data CWT, exhibit high dimensionality and complexity. To address this, the CWT-PLS method (Li et al., 2022) was used to select sensitive wavelet features for cotton wilt detection. PLS regression identified uncorrelated latent variables in high-dimensional wavelet coefficient data, reducing collinearity and avoiding overfitting or underfitting. The predictive accuracy of PLS regression was evaluated by optimizing the number of latent variables. Projection Variable Importance (VIP) values were calculated for each wavelength, with higher VIP values indicating greater importance in the CWT-PLS model. Wavelet features with higher VIP values were selected as they contained key spectral information and improved detection performance compared to the full feature set. Random Forest (RF) was used for feature selection of spectral indices. RF calculated the Out-of-Bag (OOB) error for each decision tree, introduced random noise to OOB features, and recalculated the error. Features causing a larger decrease in OOB accuracy after noise addition were identified as having a greater impact on prediction results.

The selected features constituted DSSFs for cotton leaf wilt detection. Feature selection methods, including ANOVA, PLS regression, and Random Forest, were implemented using the scikit-learn Python library (available at https://github.com/scikit-learn/scikit-learn). Finally, the Variance Inflation Factor (VIF) was used to test multicollinearity among wavelet features, and features with low multicollinearity were retained.

#### 2.4.3 Construction of classification model

Two types of cotton leaf Verticillium wilt detection were implemented, including the classification between healthy and infected leaves and the five grades of disease severity of damage. The classification methodology for distinguishing healthy and infected leaves was developed using DSSFs from samples across various DAIs in two experiments. This study employed machine learning methods coupled with wrapper feature selection (ML-FS), where the performance of machine learning classification models served as the discriminator for feature selection.

The Sequential Forward Floating Selection (SFFS) algorithm was chosen as the wrapper feature selection method, providing suboptimal solutions for computationally intensive exhaustive searches. The three types of spectral features selected from filter-based feature selection were set as the initial feature set F, and the feature subset began as an empty set $X_{0}$ . Then, SFFS iteratively selected features $x^{+}$ and added them to the feature subset $X_{k}$ to form $X_{k+1}$ , optimizing the feature function J(X) . In the context of wrapper feature selection, J(X) represented the performance of the target machine learning models on the given feature set. This process was repeated until the number of features (k) reached a preset value. The above process can be represented by Equation 1 and Equation 2.


(1) $$x^{+}=argmax\;J\left(X_{k}\;+\;x\right)$$

(2) $$X_{k+1}=X_{k}\;+\;x^{+}$$

where $x\in F-X_{k}$ . Finally, the corresponding feature subset $X_{k}$ was selected as the optimal feature combination, and the classification accuracy corresponding to this optimal feature combination was obtained.

This study employed commonly used machine learning models, such as LR, KNN, and SVM, for cotton Verticillium wilt detection, coupled with wrapper feature selection (Tian et al., 2021; Yang et al., 2024). LR uses a logistic function (sigmoid function) to map the output of linear regression to the range [0, 1]. The model is optimized using the maximum likelihood method for parameter estimation. KNN partitions the feature vector space using training data and determines the category of a new sample based on the categories of its K nearest neighbors. This study used Euclidean distance for distance measurement in KNN. SVM constructs an optimal class-separation hyperplane to maximize the margin between classes using a small number of support vectors (training samples). A linear kernel function was chosen as the decision function for SVM. During the feature selection process, 10-fold cross-validation was used to evaluate the accuracy of the classification models (LR, KNN, and SVM). For the classification of cotton leaf severity of damage and canopy incidence, the methodology was developed directly using the selected disease-specific spectral features (DSSFs) from filter-based feature selection, incorporating samples across all DAIs from both experiments. The same classification methods (LR, KNN, and SVM) were applied. The performance of the severity classification models was assessed using the classification accuracy of different severity grades of damage. All machine learning methods were implemented using the scikit-learn Python library, with wrapper feature selection provided by the mlxtend package (available at https://github.com/rasbt/mlxtend). The dataset was split into a training set (80%) and a testing set (20%). The training set was used to train the models, while the testing set evaluated model performance. Performance was evaluated using 10-fold cross-validation. The training set was divided into 10 subsamples, with one subsample retained as validation data and the remaining nine used for training. This process was repeated 10 times, with each subsample validated once. The average of the 10 validation results was taken as the final evaluation metric. The performance of the wilt diseasedetection models was evaluated using accuracy and F1-score (Li L. et al., 2024),calculated according to Equation 3-6 as follows:


(3) $$Accuracy=\frac{TP+TN}{TP+FP+FN+TN}$$

(4) $$Precision=\frac{TP}{TP+FP}$$

(5) $$Recall=\frac{TP}{TP+FN}$$

(6) $$F1=2\times \frac{Precision\times Recall}{Precision+Recall}$$

where TP is True Positive, FP is False Positive, TN is True Negative, and FN is False Negative.

## 3 Experimental results and analysis

### 3.1 Assessment of leaf biochemical parameters following *Verticillium dahliae* inoculation

**Figure 3:**
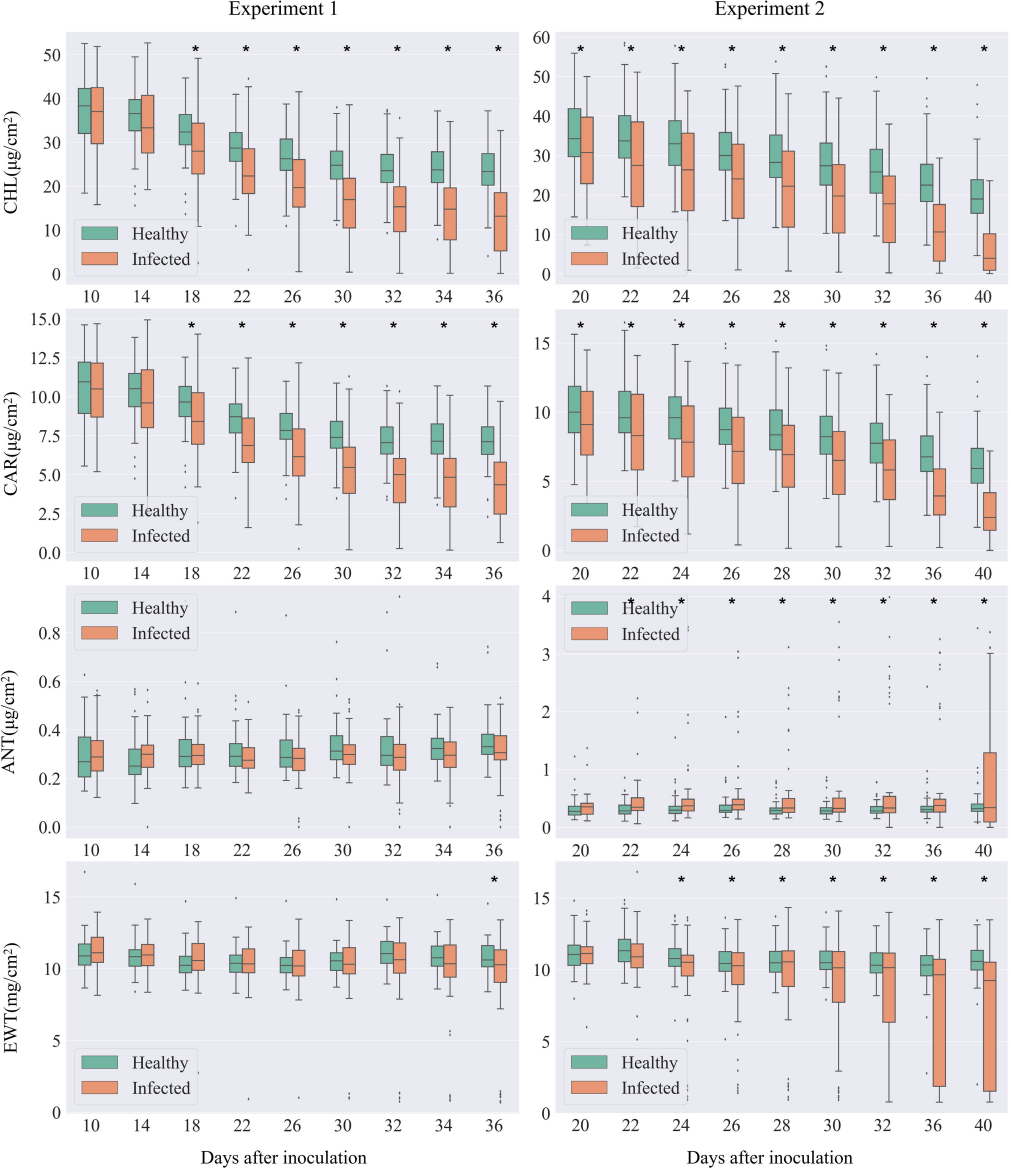
Comparison of leaf biochemical parameters between the infected and healthy leaves. The left panel shows data from Experiment 1, while the right panel presents data from Experiment 2. In each boxplot, the top edge, black line, and the bottom edge of the box represent the upper (Q3), median (Q2), and lower (Q1) quartile, respectively. The whiskers represent the maximum (Q3 + 1.5 × IQR) and minimum (Q1–1.5×IQR) valid values defined by inter-quartile ranges (IQR = Q3-Q1). The dots outside the boxplot represent outliers. Asterisks at the top of the pairs of box plots indicate significant differences in biochemical variables between healthy and infected samples, as determined by Student's t-test.

Compared to healthy leaves, cotton leaves infected with *V. dahliae* exhibited dynamic changes in biochemical parameters following inoculation (**Figure 3**). These changes aligned with the typical symptomatology of Verticillium wilt.

Levels of chlorophyll and carotenoids, key photosynthetic pigments, demonstrated the most rapid response to infection. Both pigments consistently declined as the disease progressed, with significant reductions observed from Day After Inoculation (DAI) 18.

Changes in anthocyanins and water content were slightly delayed, with no significant differences in the early stages of the disease. However, variability became evident in the middle to late stages. As the disease progressed, the rate of change in these parameters accelerated, showing strong differences in the late stages. A significant reduction in water content was observed from DAI 22. A marked increase in anthocyanins was noted from DAI 24. Due to varying disease progression rates among individual leaves, some leaves with faster symptom development appeared as outliers in the box plot compared to others. In summary, our findings demonstrate that cotton leaves infected with *V. dahliae* undergo significant biochemical changes, particularly in photosynthetic pigments and water content, reflecting the physiological impact of Verticillium wilt.

### 3.2 Spectral response patterns of cotton leaves post infection

**Figure 4:**
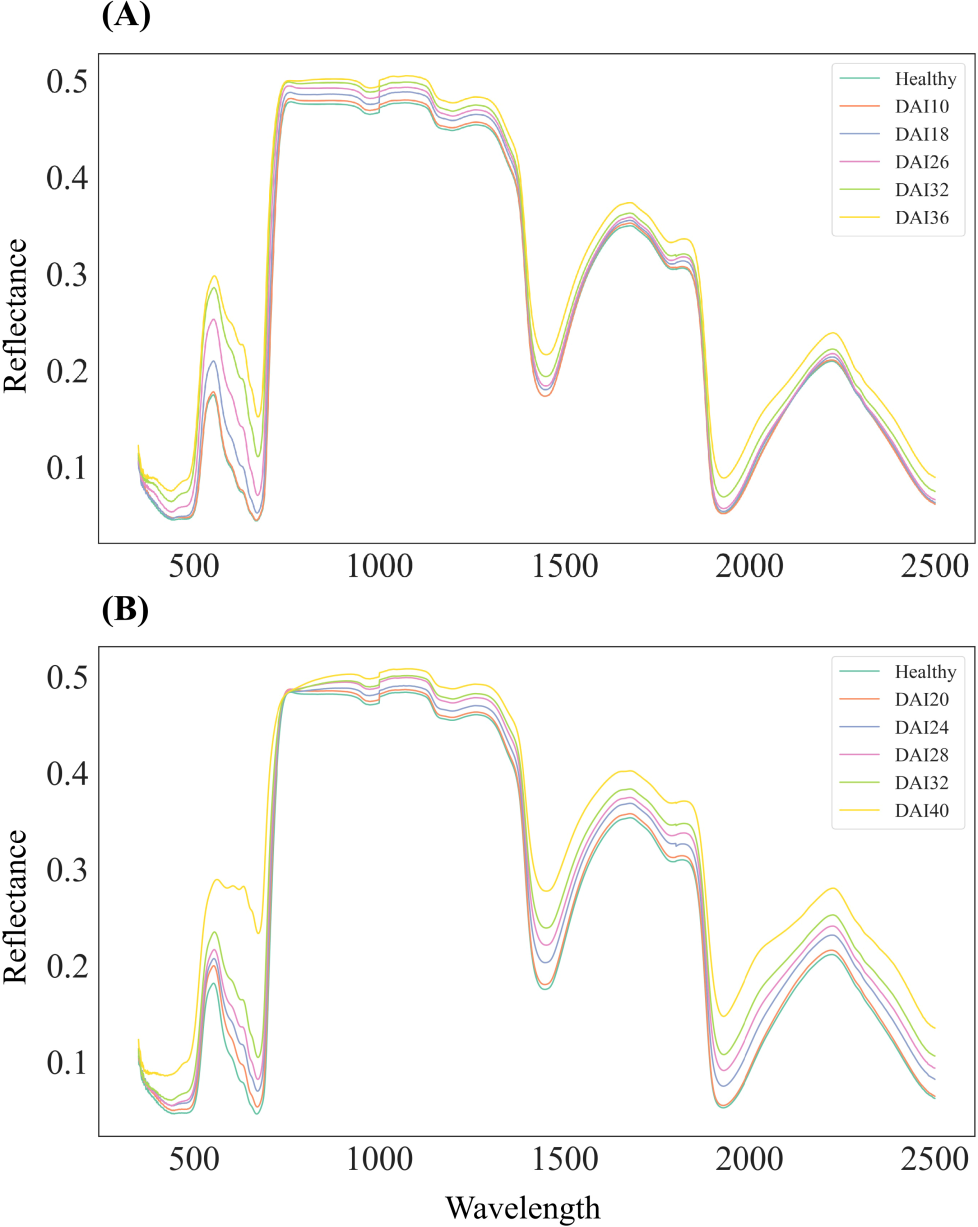
Mean leaf reflectance of healthy and infected leaves across different days after infection (DAIs), spanning wavelengths from 350 to 2500 nm. **(A)** Leaf reflectance data collected on days 10, 18, 26, 32, and 36 DAI in Experiment 1. **(B)** Leaf reflectance data collected on days 20, 24, 28, 32, and 40 DAIs in Experiment 2. The colored lines represent the reflectance of specimens at different DAIs.

Overall, the spectral response of cotton leaves infected with *V. dahliae* showed an increasing trend in reflectance across the entire wavelength range (350–2500 nm) as the disease progressed. Experiment 1 primarily covered the asymptomatic and early stages of infection, while Experiment 2 focused on the early to mid-stages (**Figure 4**). During the asymptomatic and early stages in Experiment 1, the spectral response was relatively weak (**Figure 4A**), but a significantly stronger response was observed in the early to mid-stages of Experiment 2 (**Figure 4B**). In the visible light range (400–680 nm), dominated by photosynthetic pigments such as chlorophyll and carotenoids, the response to infection was rapid and sensitive, with a gradual weakening of absorption in the green and red regions and a consistent upward trend in reflectance that continued into the mid and late stages. In contrast, the near-infrared (750–1350 nm) and shortwave infrared (1350–2500 nm) ranges showed weaker reflectance changes during the asymptomatic stage but exhibited a more pronounced upward trend as DAI increased. This was particularly evident in the water absorption valleys around 1450 nm and 1950 nm, where spectral absorption weakened significantly in the later stages, mirroring the patterns observed in the visible range. These findings indicate that the absorption characteristics of photosynthetic pigments and water are significantly reduced in infected leaves, aligning with the biochemical changes shown in **Figure 3**, where chlorophyll, carotenoids, and water content decreased as DAI increased.

### 3.3 Selection of sensitive spectral features for cotton Verticillium wilt

**Figure 5:**
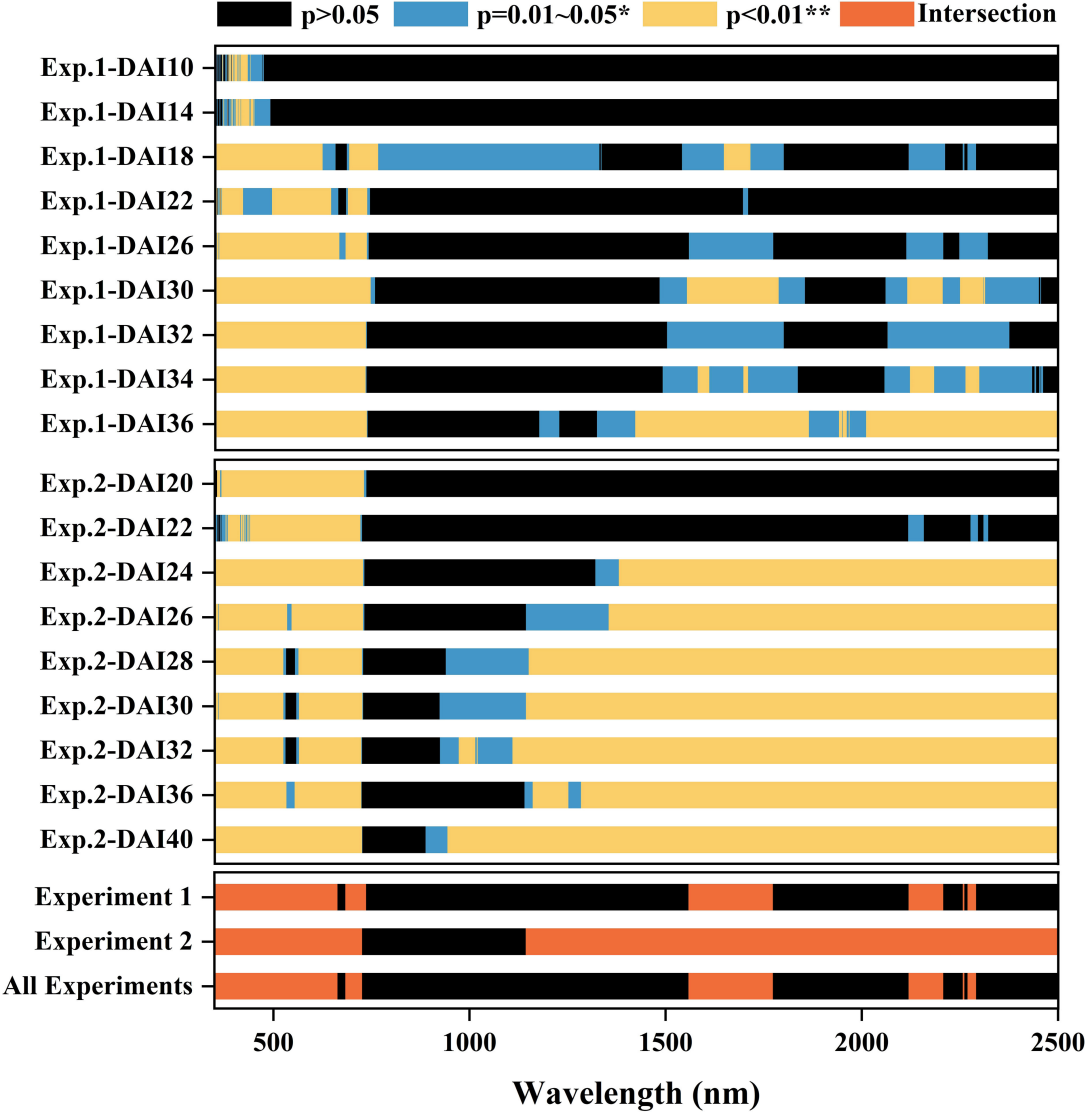
ANOVA result of reflectance spectra of the leaves collected from Experiment 1 and in Experiment 2. The intersection of sensitive bands identified from both datasets is highlighted. The X-axis has spectral wavebands from 350 to 2500 nm. The patches in red represent the intersection of the most sensitive features.

Significant reflectance features for both healthy and infected leaves were identified using filter feature selection based on AVOVA (**Figure 5**). In Experiment 1, ANOVA analysis revealed that sensitive spectral bands for cotton Verticillium wilt primarily resided within the 350–735 nm visible light range, especially during the asymptomatic to early stages. At the asymptomatic stage, a few bands began to show significant differences, with substantial differences emerging around 450 nm and 700 nm from DAI 16. As the disease progressed, significant differences became more pronounced across the entire visible light range, with stable sensitive spectral features identifiable between DAI 26 and DAI 36. In the shortwave infrared range (SWIR), no significant differences were observed during the asymptomatic stage, but differences emerged in later stages. Both experiments showed stable sensitive spectral features in the mid to late stages. The intersection of ANOVA results confirmed that the visible light range exhibited sensitive spectral features for differentiating healthy and infected leaves early on, with stable differences as the disease progressed. Although no significant differences were detected in the SWIR during the asymptomatic stage, significant distinctions aligned with water absorption characteristics in later stages. Consequently, the red (665 nm) and green (560 nm) bands were selected to reflect pigment responses, while the SWIR water absorption ranges (1450–1850 nm and 2000–2500 nm) were chosen to reflect changes in water content. Based on ANOVA scores, 560 nm, 665 nm, 1610 nm, and 2185 nm were identified as sensitive spectral bands for cotton Verticillium wilt at the leaf scale.

**Figure 6:**
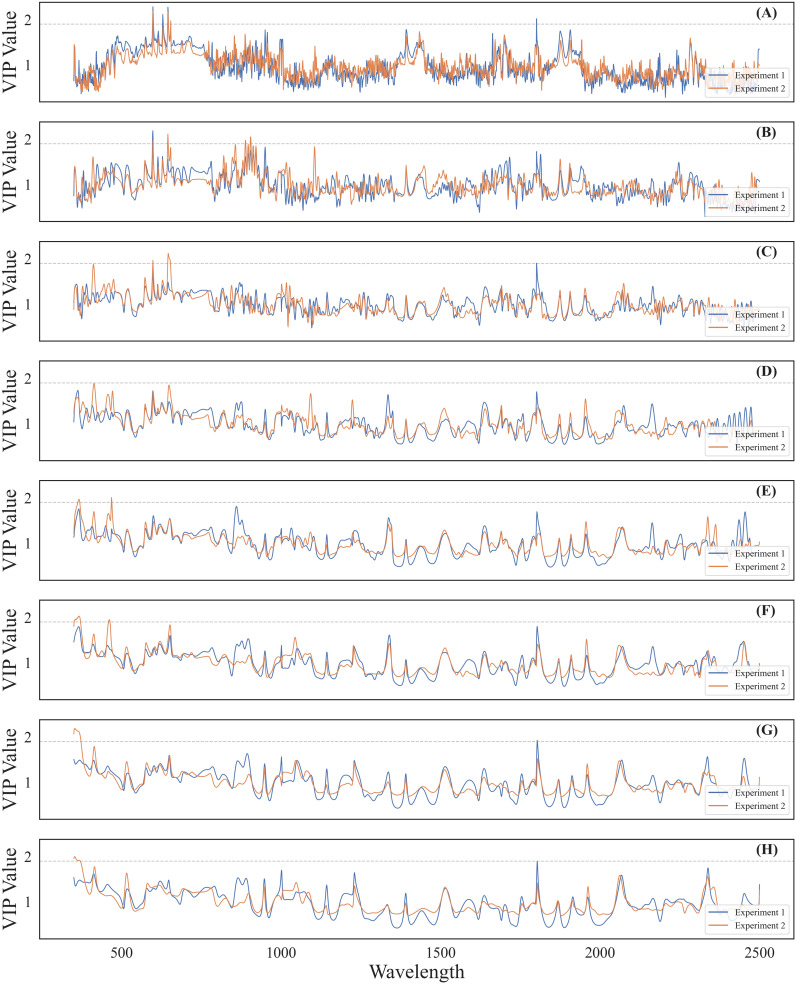
VIP values over observed wavelengths of the CWT-PLS model for incidence and severity detection of cotton Verticillium wilt in different transformation scales. The images **(A-H)** from top to bottom, correspond to transformation scales 2 to 16, respectively. The gray dashed line in each image represents a VIP threshold of 1.9.

Sensitive wavelet features for cotton leaf Verticillium wilt detection were selected by calculating VIP values following PLS regression using data from both experiments (**Figure 6**). The VIP value curves for wavelet features showed similar trends across the spectral range in both experiments. As the transformation scale increased, VIP values decreased, resulting in smoother curves due to the filtering of high-frequency signals by CWT. A VIP threshold of 1.9 was applied to filter potential sensitive wavelet features, followed by manual selection to confirm DSSFs. Peaks in VIP values were predominantly concentrated in the visible light and SWIR ranges across different transformation scales. Ultimately, 13 wavelet features were identified as DSSFs for cotton leaf Verticillium wilt detection (**Table 1**).

**Table 1:** Summary of the selected disease-specific spectral features (DSSFs) for cotton Verticillium wilt.

Feature type	Feature number	Feature name
Spectral reflectance	F1	R_560_*
	F2	R_665_*
	F3	R_1610_
	F4	R_2185_
Wavelet coefficients	F5	W_2-598_*
	F6	W_2-629_*
	F7	W_2-645_*
	F8	W_2-1801_
	F9	W_4-598_*
	F10	W_4-645_*
	F11	W_4-904_*
	F12	W_6-646_*
	F13	W_6-1801_
	F14	W_10-468_*
	F15	W_12-366_
	F16	W_14-353_
	F17	W_14-1803_
Spectral index	F18	Car_rededge*
	F19	PRI*
	F20	PSSRc*
	F21	PSRI*
	F22	NDII
	F23	LIC3*
	F24	HI_2014*
	F25	mARI*
	F26	ARI*
	F27	BF5*

The feature type column is the type of spectral features. The feature number column is the number of spectral features used to refer to the corresponding spectral features in subsequent sections. The feature name column is the name of the selected spectral feature, where spectral reflectance is represented by R with subscripts as bands, wavelet features are represented by W with subscripts as transform scales and bands, and spectral indices are detailed in **Supplementary Table 1**. The asterisk indicates that this feature has also been applied in the severity assessment models of cotton Verticillium wilt at the canopy scale.

**Figure 7:**
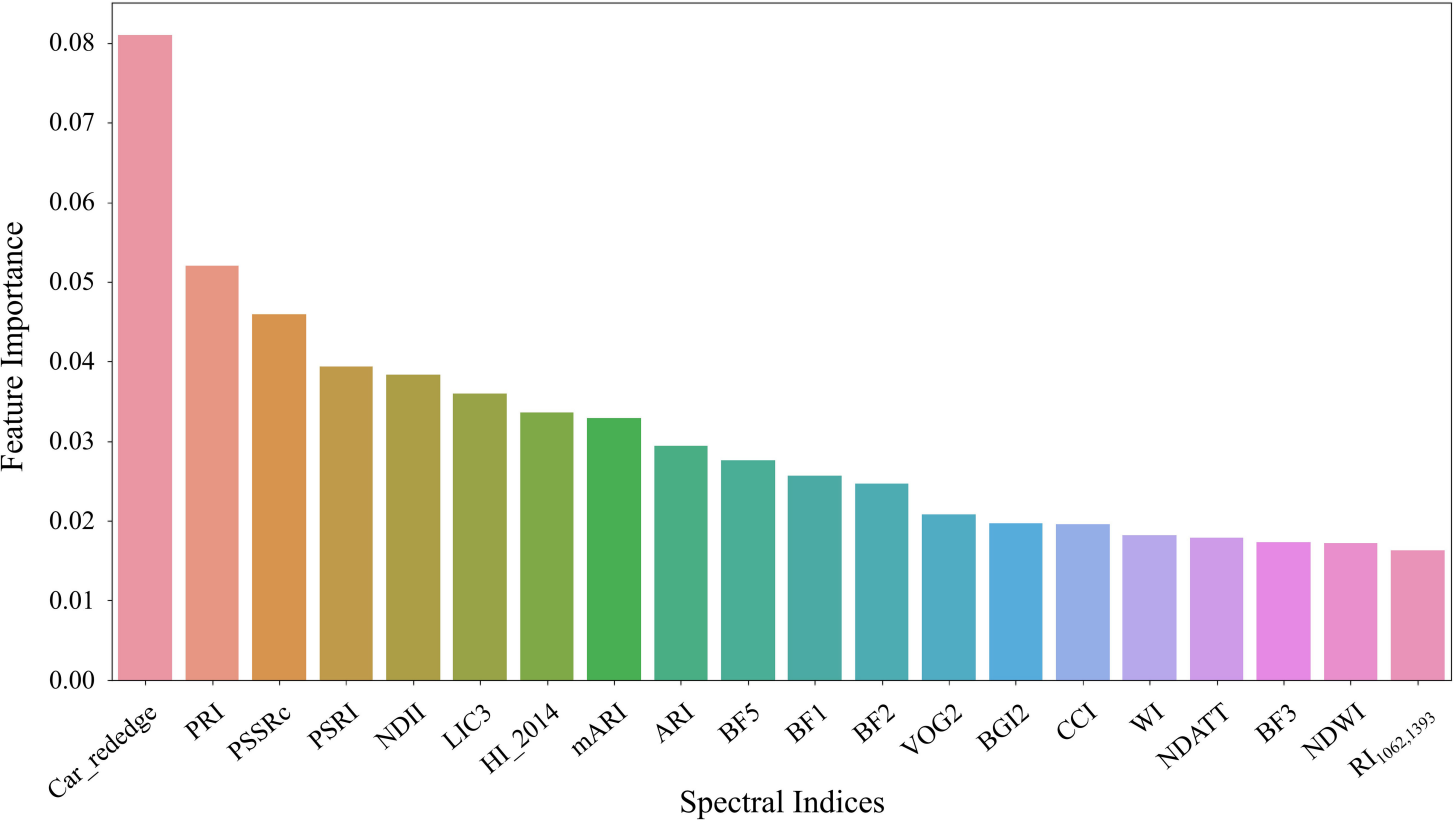
The top 20 spectral indices ranked by feature importance. Random forest analysis was conducted using data from both Experiment 1 and Experiment 2 to determine the significance of each index.

All 92 candidate spectral indices (**Supplementary Table 1**) were ranked by feature importance using a random forest approach based on data from both experiments. The top 20 indices with the highest importance included Car_rededge, PRI, PSSRc, PSRI, NDII, LIC3, HI_2014, mARI, ARI, BF5, BF1, BF2, VOG2, BGI1, CCI, WI, MDATT, BF3, NDWI, and RI1062_1393 (**Figure 7**). These indices were primarily related to photosynthetic pigments, plant stress, and water content. Specifically, Car_rededge, PRI, PSSRc, VOG2, CCI, and MDATT were linked to chlorophyll and carotenoids, while mARI, ARI, PSRI, and HI_2014 were associated with plant stress. Indices such as NDII, WI, NDWI, and RI1062_1393 were related to water content. Additionally, RGB color indices (LIC3, BF5, BF1, BF2, BGI1, and BF3) showed relationships with photosynthetic pigments. The top 10 sensitive spectral indices were selected for cotton leaf Verticillium wilt detection.

Based on the feature selection results from both experiments, 27 features were ultimately identified as DSSFs for cotton Verticillium wilt detection, including spectral reflectance and wavelet coefficients (**Table 1**). This comprehensive analysis highlights the importance of specific wavelengths and features in monitoring plant health and disease progression, providing critical insights for the early detection of cotton Verticillium wilt.

### 3.4 Incidence detection of cotton leaf Verticillium wilt coupled with wrapper feature selection

To enhance the selection of spectral features, we employed the SFFS as a wrapper feature selection algorithm. This method refined the preliminary selection of spectral features from infected and healthy cotton leaves at different infection stages, identified through filter-based feature selection. The selected features were then used as input data to construct classification models for cotton Verticillium wilt using three machine learning methods: LR, KNN, and SVM. The optimal feature combinations and classification performance of each model at different DAIs were evaluated (**Table 2**).

**Table 2:** Performance of the ML-FS techniques in accuracy for classifying healthy and infected samples by 10-fold cross-validation, including Logistic Regression, K-Nearest Neighbor, and Support Vector Machine.

Experiment	Day After Inoculation	Logistic Regression		K-Nearest Neighbor		Support Vector Machine	
		Optimal Feature Combination	Mean Accuracy	Optimal Feature Combination	Mean Accuracy	Optimal Feature Combination	Mean Accuracy
Experiment 1	10	F2,F1,F3,F4	63.33%	F5,F7,F9,F22	**65.00%**	F2,F1,F3,F4	63.33%
	14	F2,F1,F3,F4	64.17%	F5,F6,F9,F9	**72.50%**	F2,F1,F3,F4	63.33%
	18	F2,F1,F18,F20	65.00%	F7,F6,F9,F24	**78.33%**	F2,F1,F3,F4	63.33%
	22	F2,F1,F3,F4	**67.50%**	F1,F5,F7,F6	**67.50%**	F2,F1,F3,F4	65.00%
	26	F2,F1,F3,F4	65.83%	F2,F5,F7,F12	**75.00%**	F2,F1,F3,F4	63.33%
	30	F2,F18,F20,F27	**72.50%**	F7,F10,F17,F22	**72.50%**	F2,F1,F20,F26	71.67%
	32	F2,F1,F18,F20	69.17%	F5,F7,F19,F26	**77.50%**	F2,F3,F20,F25	68.33%
	34	F2,F20,F25,F27	76.67%	F3,F4,F23,F26	**81.67%**	F2,F1,F20,F26	75.00%
	36	F2,F1,F20,F26	75.83%	F5,F12,F17,F23	**77.50%**	F2,F20,F25,F26	76.67%
Experiment 2	20	F4,F5,F26,F27	67.50%	F2,F5,F18,F23	**74.17%**	F2,F18,F20,F26	65.83%
	22	F2,F1,F3,F26	**72.50%**	F7,F6,F8,F19	70.83%	F2,F1,F20,F26	**72.50%**
	24	F2,F1,F18,F27	70.00%	F2,F15,F23,F25	**75.00%**	F2,F1,F3,F26	69.17%
	26	F2,F1,F3,F26	70.00%	F2,F20,F21,F22	**72.50%**	F2,F1,F20,F26	67.50%
	28	F1,F5,F18,F25	70.83%	F5,F7,F6,F27	**72.50%**	F2,F18,F20,F26	70.00%
	30	F2,F18,F20,F27	72.50%	F5,F16,F21,F23	**73.33%**	F2,F1,F3,F20	69.17%
	32	F2,F1,F18,F20	**74.17%**	F5,F7,F8,F23	70.83%	F2,F1,F18,F20	71.67%
	36	F2,F1,F3,F20	**77.50%**	F5,F7,F23,F27	76.67%	F1,F3,F4,F20	76.67%
	40	F2,F1,F3,F20	83.33%	F4,F5,F7,F21	**85.83%**	F1,F3,F20,F27	83.33%

Bold indicates the best predictor for each observation period.

Results from the two experiments revealed that the machine learning classification model based on wrapper feature selection achieved a classification accuracy slightly above 60% during the asymptomatic stage. As the disease progressed, the model’s accuracy improved incrementally, ultimately exceeding 85%. Notably, the KNN model consistently outperformed LR and SVM across all disease stages. In Experiment 1, KNN achieved the highest accuracy of 77.50%, while in Experiment 2, it reached 85.83%. The superiority of KNN was particularly evident during the asymptomatic stage, where its performance significantly exceeded that of LR and SVM. As the infection progressed, the performance gap between the three algorithms narrowed. While SVM initially showed lower accuracy during the asymptomatic and early stages, its accuracy at the final DAI in both experiments reached 76.67% and 83.33%, closely matching or even surpassing the other algorithms in some cases. Additionally, the OFC selected by the three algorithms varied slightly across different DAIs and models.

**Figure 8:**
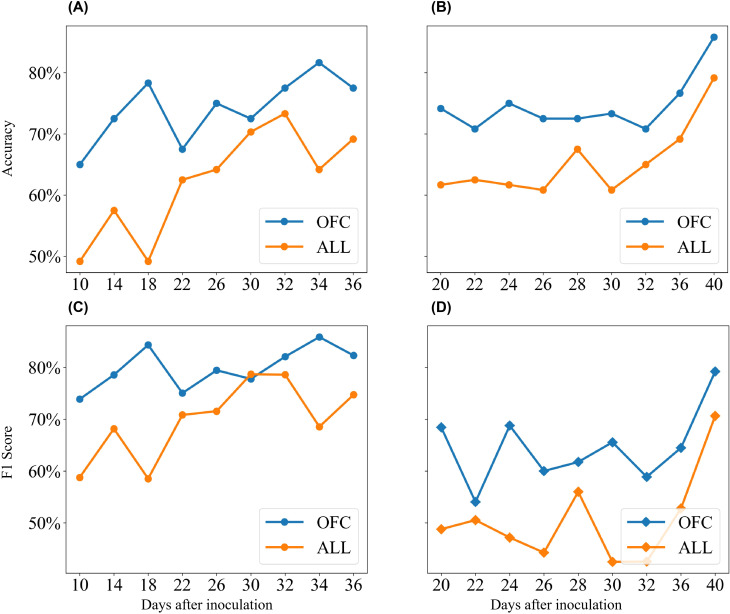
Comparison of classification accuracy and F1-score between standard KNN and KNN with wrapper feature selection. **(A)** Results of accuracy from Experiment 1. **(B)** Results of accuracy from Experiment 2. **(C)** Results of F1-score from Experiment 1. **(D)** Results of F1-score from Experiment 2. "ALL" represents models trained with all features, while "OFC" indicates models constructed using the ML-FS algorithm.

As the disease progressed, both the traditional KNN model and the KNN model using OFC showed gradual increases in classification accuracy and F1 score (**Figure 8**). The KNN model using OFC achieved an accuracy exceeding 85% and an F1 score of 82%, while the model using all features achieved an accuracy close to 80% and an F1 score of approximately 75%. Across all observations, the KNN model enhanced by wrapper feature selection consistently outperformed the standard KNN algorithm. This advantage was particularly pronounced in the early stage of Experiment 1, where the wrapper-based KNN model achieved 29% higher accuracy and 25% higher F1 score compared to the standard KNN. Although the performance gap narrowed during the mid-stage of the disease, the wrapper-based KNN maintained a clear advantage. Overall, the KNN model, enhanced by wrapper feature selection, demonstrated superior accuracy in detecting cotton leaf Verticillium wilt, achieving over 85% accuracy and 82% F1 score as the disease progressed, significantly outperforming LR and SVM models, especially during the asymptomatic stage.

### 3.5 Severity detection of cotton leaf damage by Verticillium wilt

**Figure 9:**
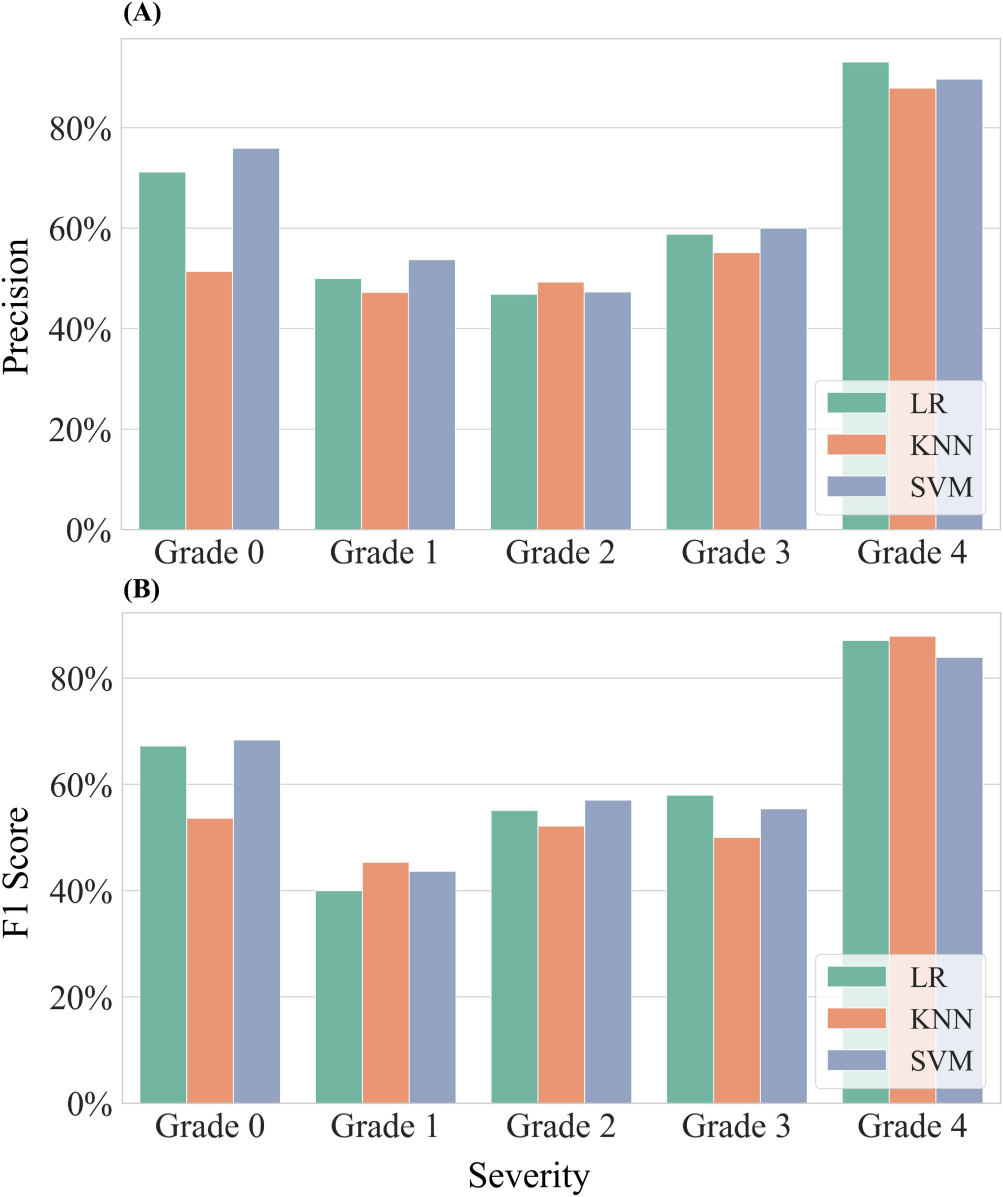
Assessment in precision for classifying different severities of samples. Three models—Logistic Regression (LR), K-Nearest Neighbors (KNN), and Support Vector Machine (SVM)—were employed for this evaluation. **(A)** Results of precision for all samples. **(B)** Results of F1-Score for all samples.

Using the selected DSSFs from the first stage of feature selection as input data, along with the corresponding severity grades of damage as labels, we constructed classification models to assess the severity of cotton leaf damage by Verticillium wilt. The same three machine learning methods—LR, KNN, and SVM—were employed, and the classification performance for different severity grades of damage was evaluated (**Figure 9**).

Analysis of samples from both experiments revealed that the precision of the machine learning models improved as the severity grade of infected cotton leaves increased. The models achieved their highest precision for the most severe grade (Grade 4), with precision ranging from 87.88% to 93.10%. In contrast, precision was lower and more comparable for the other grades: Grade 1 (47.22%–53.75%), Grade 2 (46.82%–49.26%), and Grade 3 (55.17%–60.00%). For Grade 0, classification performance varied, with the SVM model achieving a maximum precision of 75.93%, while the KNN model recorded only 51.39%. The LR and SVM models showed similar precision across all severity grades, with differences not exceeding 5%. Although the KNN model was generally less accurate than LR and SVM, it performed slightly better for Grade 2. The F1 scores exhibited similar trends to precision across severity grades. The highest F1 scores from Grade 0 to Grade 4 were 68.33%, 45.33%, 57.05%, 57.97%, and 87.88%, respectively.

### 3.6 Incidence detection of cotton Verticillium wilt at canopy scale

Furthermore, this study explored the potential for upscaling the application of DSSFs selected at the leaf scale to the canopy level. Using field hyperspectral data collected by UAV, we investigated the detection of cotton canopy Verticillium wilt incidence. Due to differences in spectral ranges between the leaf-scale spectrometer and the airborne hyperspectral camera, sensitive spectral features compatible with UAV data were filtered from the DSSFs identified in the first stage of feature selection (**Table 1**). These filtered features were used as input data for constructing an incidence detection model at the canopy scale.

A total of 19 DSSFs matching the UAV hyperspectral data were used as input for the canopy-scale incidence detection model. On different Days After Sowing (DASs), the accuracy of the model was evaluated using three machine learning methods (**Table 3**). Over the observation period, the classification accuracy of the models increased from approximately 50% at DAS 52 to over 90% at DAS 101, while F1 scores improved from slightly above 60% to over 95%. Notably, from DAS 87 to DAS 101, the performance of all three models was highly consistent, with classification accuracy exceeding 90% and F1 scores above 95%, demonstrating effective and stable identification of cotton canopy Verticillium wilt during this period. Between DAS 59 and DAS 80, the KNN model outperformed the others, showing faster improvement in classification accuracy before converging with the performance of the other models.

**Table 3:** Performance of the incidence detection models of cotton canopy Verticillium wilt by 10-fold cross-validation, including Logistic Regression, K-Nearest Neighbor, and Support Vector Machine.

Day after Sowing	Logistic Regression		K-Nearest Neighbor		Support Vector Machine	
	Mean Accuracy	Mean F1 Score	Mean Accuracy	Mean F1 Score	Mean Accuracy	Mean F1 Score
52	49.00%	61.59%	**52.00%**	60.36%	51.00%	**64.84%**
59	**59.50%**	72.89%	**59.50%**	64.36%	**59.50%**	**74.60%**
66	58.50%	65.78%	**68.50%**	**70.31%**	58.50%	66.68%
73	61.50%	75.19%	**71.00%**	**76.26%**	65.50%	74.25%
80	**72.00%**	**83.70%**	71.00%	79.88%	**72.00%**	**83.70%**
87	**91.50%**	**95.55%**	88.50%	94.12%	**91.50%**	**95.55%**
94	**92.50%**	**96.09%**	90.00%	94.99%	**92.50%**	**96.09%**
101	**93.00%**	**96.36%**	92.50%	95.80%	**93.00%**	**96.36%**

Bold indicates the best predictor for each observation period.

## 4 Discussion

### 4.1 Physiological explanation of the spectral response of cotton Verticillium wilt

*V. dahliae* induces symptoms in cotton leaves by colonizing the xylem, leading to xylem blockage and the secretion of toxins (Yadeta and J Thomma, 2013; Chen et al., 2016; Zhang et al., 2022). Following infection, cotton undergoes a series of physiological reactions, and changes in various biochemical parameters can trigger significant responses within specific spectral ranges (Yang et al., 2024). Due to the complexity of cotton’s physiological response to *V. dahliae*, there may be an imbalance in the spectral features selected from the visible-near infrared (VNIR) and SWIR ranges.

This study reveals that the temporal response pattern of leaf biochemical parameters to Verticillium wilt infection aligns with the physiological response of cotton to the disease. In the early stages of infection, only a small amount of *V. dahliae* infects the cotton plants, which is insufficient to block the xylem. As a result, there are no significant differences in biochemical parameters between infected and healthy leaves. However, as the fungus grows and the xylem becomes blocked, photosynthetic pigments (chlorophyll and carotenoids) in cotton plants respond rapidly to the infection, and their response remains stable and continuous until the end of the observation period (**Figure 3**). By the mid to late stages, anthocyanins and water content begin to show responses to the infection, with more intense changes than those observed in photosynthetic pigments, indicating a severe progression of the disease.

The rapid proliferation of *V. dahliae* damages the internal structure of leaves and disrupts the transport of water and nutrients (Tian and Kong, 2022). VNIR spectral features are highly sensitive to changes in the internal structure of leaves and pigment content (Jacquemoud et al., 2009; Ustin et al., 2009). CWT is widely recognized for its ability to detect subtle spectral signals (Lin et al., 2021), and the extracted small wavelet features are highly interpretable (Li et al., 2021). These techniques highlight subtle changes in the local spectrum of cotton infected with Verticillium wilt, significantly improving the detection accuracy of the biophysical dynamic characteristics of infected leaves (Zhang et al., 2019).

The biochemical response of cotton to Verticillium wilt infection, based on physiological mechanisms, can guide the selection of disease-specific spectral features (DSSFs). The sensitivity of numerous spectral features in the VNIR region during feature selection (**Figure 5**) suggests that pigment content may respond rapidly to pathogen infection. Spectral characteristics in the SWIR region also exhibit sensitivity to Verticillium wilt, particularly in the later stages of infection. Correspondingly, the water content of infected cotton leaves shows a significant decrease in the late stages (**Figure 3**), indicating that water stress caused by Verticillium wilt occurs relatively later. Specifically, the 665 nm spectral band selected during sensitive spectral feature selection coincides with the chlorophyll absorption center, suggesting that this band can effectively capture changes in chlorophyll content due to Verticillium wilt infection. The selected wavelet features (W4-613, W4-629) characterize changes in the spectral shape near the yellow range, primarily influenced by the overlapping absorption of several pigments, particularly chlorophyll and anthocyanins (Féret et al., 2017). Most of the selected SIs are also related to chlorophyll and carotenoids (Car_rededge, PRI, PSSRc, VOG2, CCI, MDATT, mARI, ARI, PSRI, and HI_2014), which can be explained by the strong correlation between chlorophyll and carotenoid content in crop leaves (Féret et al., 2011).

This study, through dense sampling, reveals the physiological and spectral responses of cotton plants to Verticillium wilt infection at the leaf level. Determining disease-specific spectral features based on the physiological mechanisms of specific disease stress is crucial (Zarco-Tejada et al., 2018). The delayed response of anthocyanins and water content in detecting various disease stresses suggests that traditional methods relying on these indicators may not be sufficient for early detection of cotton Verticillium wilt (Morel et al., 2018). Instead, photosynthetic pigments such as chlorophyll and carotenoids should be prioritized as sensitive indicators. This study analyzes the universal spectral response patterns of cotton to Verticillium wilt stress based on spectral data from different cotton varieties and selects DSSFs suitable for various cotton varieties. There are slight differences in the spectral responses of different cotton varieties to Verticillium wilt stress. Therefore, future research should focus on exploring sensitive spectral features with variety specificity to enhance the accuracy of detecting Verticillium wilt in different cotton varieties.

### 4.2 Advantages of machine learning methods based on feature selection

Compared to traditional machine learning methods that utilize all features for classification, the wrapper-based feature selection approach adopted in this study enhances the performance of the classification model by identifying the optimal feature combination. As DAI increases, the classification accuracy of both methods improves significantly, but the ML-FS (Machine Learning with Feature Selection) method consistently achieves higher accuracy than traditional methods (**Figure 8**). This demonstrates that models constructed with OFC not only reduce computational costs but also provide superior classification accuracy compared to models trained using all features. Recent studies have increasingly focused on identifying optimal features to improve classification performance (Liu et al., 2019; Hornero et al., 2020; Tian et al., 2021; Yang et al., 2024). By optimizing the combination of sensitive spectral features, it is possible to effectively amplify the weak spectral signals generated by the physiological response of plants to pathogen stress during the early stages of disease. In terms of computational efficiency, the ML-FS method has more advantages than methods based on radiative transfer models. Due to the need to use all hyperspectral data for model inversion of biochemical parameters, the radiative transfer model requires a longer calculation time, limiting its application in disease detection (Hornero et al., 2020). On the contrary, feature selection algorithms greatly improve computational efficiency by selecting the optimal combination from hyperspectral data while retaining useful information (Huang et al., 2019).

Another advantage of the ML-FS method is its ability to ensure the rationality and interpretability of the OFC obtained through feature selection. Previous studies (Garcia Furuya et al., 2021; Poblete et al., 2021; Yu et al., 2022) have employed large numbers of unselected features as inputs for machine learning to achieve effective disease detection, but the specificity of these features for particular diseases remains unclear. This study, however, identifies sensitive features for cotton Verticillium wilt with high information content through feature selection, providing insights into the response mechanisms of these features to disease occurrence. Filter-based feature selection methods evaluate features based on general performance metrics such as target correlation, autocorrelation, and divergence without considering specific models. The advantages of this approach include low computational cost and effective avoidance of overfitting. However, its drawback is that it does not account for the specific learners to be used in the future, which may weaken the learners’ fitting ability. This method is well-suited for applications requiring strong universality, such as spectral reflectance. PLS offers advantages such as handling highly collinear data, suitability for small-sample, large-feature problems, and insensitivity to outliers. However, its disadvantages include high computational cost, poor interpretability, and limited applicability. Continuous Wavelet Transform (CWT) decomposes spectra into numerous wavelet features, effectively characterizing spectral signals but potentially introducing multicollinearity, which can be mitigated by using PLS as the feature selection method.

RF excels in processing high-dimensional data without the need for dimensionality reduction, evaluating feature importance during model training, and reducing redundant features to enhance model performance. However, RF is sensitive to noisy data, lacks model interpretability, and has an opaque feature selection process. Therefore, RF is suitable for selecting a large number of vegetation indices, and the selected indices can be interpreted based on their inherent characteristics and remote sensing mechanisms.

### 4.3 Potential applications and limitations

Among the selected Disease-Specific Spectral Features (DSSFs) for cotton Verticillium wilt (**Table 1**), a significant number of features are related to photosynthetic pigments (chlorophyll and carotenoids), such as R665, ChlRE_opt, RI_708_775, PSSRa, PSSRc, PSNDb, PR, and PRIm2 (**Supplementary Table 1**). These features, originating from the visible and near-infrared (VNIR) region, demonstrate strong potential for detecting cotton Verticillium wilt due to their connection with the physiological effects induced by the disease. Photosynthetic pigments exhibit a significant response to the onset of cotton wilt disease even during the asymptomatic stage, suggesting that they can serve as effective and consistent indicators for early detection. Recent studies have confirmed the feasibility of accurately estimating leaf chlorophyll content at various scales (Gitelson and Solovchenko, 2017; Gitelson et al., 2019; Xu et al., 2019; Li et al., 2020), highlighting the transferability and potential applications of these features for early detection across different datasets, cotton varieties, and environmental conditions. Additionally, the advantages of Continuous Wavelet Transform (CWT) in quantifying plant disease severity have been well-documented (Cheng et al., 2010; Zhang et al., 2014; Shi et al., 2018b). Our results further indicate that wavelet analysis can effectively capture subtle changes in spectral shape, enhancing the detection of disease-induced signals.

This study also demonstrates that the DSSFs selected for cotton wilt disease detection exhibit potential transferability across tasks, scales, and environments. These features can be adapted for severity of damage assessment at the leaf scale and incidence detection at the canopy scale in field conditions using machine learning methods. Based on the sensitive spectral features identified for disease occurrence detection, the classification of disease severity of damage at the leaf level shows promising results. The classification accuracy for severely infected leaves reaches 93.1%, while the accuracy for mildly infected leaves ranges between 45% and 60%. This indicates that the selected spectral features possess good cross-task transferability. Furthermore, when applied to canopy-scale disease detection using UAV hyperspectral data, these features also perform well. After DAS 80, the classification accuracy of most detection models exceeds 90%, and for samples with mild early symptoms, the accuracy remains above 50%. This underscores the strong transferability of these features across environments and scales, providing a foundation for future work on early detection of cotton wilt disease. Since different severity grades of damage represent different stages of the disease, the ability to detect subtle changes at the asymptomatic stage is critical. Sensitive narrow wavebands in hyperspectral data can respond to these subtle changes, making them valuable for early disease detection (Bai and Jin, 2024). However, while the severity assessment models perform well for severely infected leaves, the detection accuracy for mildly infected leaves remains suboptimal, highlighting the limitations of the current DSSFs for early detection. Therefore, more specific spectral features tailored for early detection need to be developed from hyperspectral data.

Compared to previous studies on cotton wilt disease, the innovation of this study lies in its use of feature selection methods to identify sensitive spectral features in greenhouse environments and investigate their transferability across tasks, environments, and scales. While recent studies have begun to explore the role of feature selection in identifying sensitive features for cotton wilt disease (Yang et al., 2022; Yuan et al., 2023; Yang et al., 2024), the methods employed have often been limited in scope. This study adopts more suitable feature selection methods for different types of spectral features and identifies sensitive spectral features at the canopy scale. Previous research on cotton wilt using hyperspectral technology has primarily focused on the leaf scale, utilizing handheld hyperspectral imaging devices to collect data (Jing et al., 2009; Chen et al., 2010, 2012; Yang et al., 2022, 2024). In contrast, this study extends the application of sensitive spectral features from the leaf scale to canopy-scale in field environments, providing a more comprehensive approach to remote sensing monitoring of cotton wilt disease.

Remote sensing offers a non-destructive, large-scale, and rapid method for detecting crop diseases, particularly for early monitoring, which is crucial for minimizing crop losses and ensuring agricultural productivity. Future research should continue to explore remote sensing monitoring of cotton Verticillium wilt using multispectral and hyperspectral satellite sensors. In addition to the widely used vegetation indices and the DSSFs selected in this study, further development of new spectral indices tailored for cotton Verticillium wilt is essential. These indices could enable monitoring at multiple growth stages (leaf, canopy, and regional scales) and across different disease progression levels. The multi-temporal detection and severity assessment of leaf diseases in this study provide a solid foundation for detecting cotton wilt disease at various stages. However, more specific spectral features need to be constructed and selected from hyperspectral data to improve early detection capabilities.

It is also important to note that the spatial heterogeneity of cotton wilt disease necessitates the expansion of datasets to include different varieties, regions, and growth stages. This will enable a deeper understanding of the spatiotemporal heterogeneity of the disease and facilitate the development of remote sensing monitoring models with strong generalization capabilities. Such models are critical for formulating effective prevention and control strategies for cotton wilt disease.

Furthermore, the near-infrared region plays a significant role in detecting various plant diseases and pests (Zhang et al., 2019). However, due to the complexity of spectral responses in this region, existing literature has not fully explored the local spectral shape changes in the NIR region. By applying wavelet transform, subtle disease-specific information in the NIR region can be enhanced and effectively utilized, significantly improving the detection of weak signals induced by cotton wilt disease. This approach holds great promise for advancing early detection and monitoring of the disease.

## Data Availability

The raw data supporting the conclusions of this article will be made available by the authors, without undue reservation.

## Supplementary material

Supplementary Table 1 Shows the spectral indices (SIs) included in this study and their formulations.
