# Supplementary material for: Spectroscopic detection of cotton Verticillium wilt by spectral feature selection and machine learning methods
Source: Front Plant Sci. 2025 May 15;16:1519001. doi: 10.3389/fpls.2025.1519001 (PMC12119526; doi:10.3389/fpls.2025.1519001)
Supplement: Supplementary Table 1 — Shows the spectral indices (SIs) included in this study and their formulations. [file Table1.docx]

Supplementary Material

## Supplementary Tables

**Supplementary Table 1.** Spectral indices (SIs) included in this study and their formulations.

| Type | Index | Short | Formulation | Reference |
| --- | --- | --- | --- | --- |
| Chlorophyll | Reciprocal Reflectance | RR | $1/R_{700}$ | (Gitelson et al., 1999) |
|  | Modified Red-edge Ratio | mSR | (R_750_-R_445_)/(R_705_-R_445_) | (Sims and Gamon, 2002) |
|  | Pigment Specific Simple Ratio | PSSRa | R_800_/R_675_ | (Blackburn, 1998a) |
|  |  | PSSRb | R_800_/R_650_ | (Blackburn, 1999) |
|  | Normalized Difference Vegetation Index | NDVI | (R_NIR_-R_R_)/(R_NIR_+R_R_) | (Rouse et al., 1974) |
|  | Red-edge NDVI | mNDVI | (R_750_-R_705_)/  (R_750_+R_705_) | (Gitelson and Merzlyak, 1994; Sims and Gamon, 2002) |
|  | Green NDVI | gNDVI | (R_750_-R_G_)/(R_750_+R_G_) | (Gitelson and Merzlyak, 1996; Datt, 1998) |
|  | Pigment Specific Normalized Difference | PSNDa | (R_800_-R_675_)/  (R_800_+R_675_) | (Blackburn, 1998b) |
|  |  | PSNDb | (R_800_-R_650_)/  (R_800_+R_650_) |  |
|  |  | PSNDc | (R_800_-R_470_)/  (R_800_+R_470_) |  |
|  | Summed Reflectance Index | SRI1 | $\int_{700}^{750} {(R}/{R_{555}-1)}d$ | (Gitelson and Merzlyak, 1994) |
|  |  | SRI2 | $\int_{700}^{750} {(R}/{R_{705}-1)}d$ |  |
|  | Macc01 | Macc01 | (R_780_-R_710_)/(R_780_-R_680_) | (Maccioni et al., 2001) |
|  | The MERIS terrestrial chlorophyll ind | MTCI | (R_754_-R_709_)/(R_709_-R_681_) | (Dash and Curran, 2004) |
|  | DATT | DATT | (R_850_-R_710_)/(R_850_-R_680_) | (Datt, 1999) |
|  | Modified DATT | MDATT | (R_721_-R_744_)/(R_721_-R_714_) | (Lu et al., 2018) |
|  | Vogelmann indices | VOG1 | R_740_/R_720_ | (Vogelmann et al., 1993) |
|  |  | VOG2 | (R_734_-_747_)/(R_715_-_726_) |  |
|  |  | VOG3 | (R_734_-R_747_)/(R_715_+R_720_) |  |
|  | Gitelson & Merzlyak indices | GM1 | R_750_/R_550_ | (Gitelson and Merzlyak, 1996) |
|  |  | GM2 | R_750_/R_700_ |  |
|  | Transformed Chlorophyll Absorption in Reflectance Index | TCARI | 3×[(R700-R670)-0.2×(R700-R550)× (R700/R670)] | (Haboudane et al., 2002) |
|  | Chlorophyll Index Red Edge | CI | R_750_/R_710_ | (Haboudane et al., 2002) |
|  | Simple Ratio Pigment Index | SRPI | R_430_/R_680_ | (Penuelas et al., 1995) |
|  | Normalized Pigments Index | NPCI | (R_680_-R_430_)/(R_680_+R_430_) | (Penuelas et al., 1995) |
|  | Carter indices | CTRI1 | R_695_/R_420_ | (CARTER and Gregory, 1994) |
|  |  | CAR | R_695_/R_760_ |  |
|  | Reflectance band ratio indices | DCabCxc | R672/(R550×3R708) | (Datt, 1998) |
|  |  | NDIRCabCxc | R_860_/(R_550_×R_708_) |  |
|  | Structure-Intensive Pigment Index | SIPI | (R_800_-R_445_)/(R_800_+R_680_) | (Penuelas et al., 1995) |
|  | ChlRE opt | $ChlRE opt$ | (1/R_680-730_-1/R_780-800_)×R_755-780_ | (Féret et al., 2011) |
|  | RI_708,775_ | RI_708,775_ | R_708_/R_775_ | (Féret et al., 2011) |
|  | ND_780,712_ | ND_780,712_ | (R_780_-R_712_)/(R_780_+R_712_) | (Féret et al., 2011) |
|  | Chl_green | Chl_green | Rnir/Rgreen-1 | (Gitelson et al., 2006) |
|  | Chl_red edge | Chl_red edge | Rnir/Rred_edge-1 | (Gitelson et al., 2006) |
|  | Chlorophyll/carotenoid Index | CCI | (R_531_-R_645_)/(R_531_+R_645_) | (Gamon et al., 2016) |
| Carotenoid | Ratio Analysis of  Reflectance Spectra | RARSc | R_760_/R_500_ | (Chappelle et al., 1992) |
|  | Pigment Specific  Simple Ratio | PSSRc | R_800_/R_470_ | (Blackburn, 1998) |
|  | Modified Carotenoid  Reflectance Index | ${mCRI}_{G}$ | $(R_{510-520}^{-1}-R_{560-570}^{-1})R_{NIR}$ | (Gitelson et al., 2006) |
|  |  | ${mCRI}_{RE}$ | $(R_{510-520}^{-1}-R_{690-710}^{-1})R_{NIR}$ |  |
|  | Photochemical Reflectance Index | PRI | (R_531_-R_570_)/(R_531_+R_570_) | (Gamon et al., 1992) |
|  | Carotenoid Reflectance  Index | CRI_550_ | (1/R_510_)-(1/R_550_) | (Anatoly et al., 2002) |
|  |  | CRI_700_ | (1/R_510_)-(1/R_700_) |  |
|  |  | CRI_515,550_ | (1/R_515_)-(1/R_550_) |  |
|  |  | CRI_515,700_ | (1/R_515_)-(1/R_700_) |  |
|  |  | PNIR×CRI_700_ | (1/R_510_)-(1/R_550_)×R_770_ |  |
|  |  | PNIR×CRI_770_ | (1/R_510_)-(1/R_700_)×R_770_ |  |
|  | ${Car}_{RE opt}$ | ${Car}_{RE opt}$ | (1/R_510-530_-1/R_680-730_)×R_760-780_ | (Féret et al., 2011) |
|  | RI_530,800_ | RI_530,800_ | R_530_/R_800_ |  |
|  | ND_800,530_ | ND_800,530_ | (R_800_-R_530_)/(R_800_+R_530_) |  |
|  | Car_green | Car_green | $(R_{510-520}^{-1}-R_{560-570}^{-1})\times R_{NIR}$ | (Gitelson et al., 2006) |
|  | Car_red edge | Car_red edge | $(R_{510-520}^{-1}-R_{590-710}^{-1})\times R_{NIR}$ | (Gitelson et al., 2006) |
| Anthocyanin | Anthocyanin Reflectance Index | ARI | $R_{550}^{-1}-R_{700}^{-1}$ | (Gitelson et al., 2001) |
|  | Modified Anthocyanin  Reflectance Index | mARI | $(R_{530-570}^{-1}-R_{690-710}^{-1})\times R_{NIR}$ | (Gitelson et al., 2006) |
|  | Red:Green Ratio | RGR | R/G | (Gamon and Surfus, 1999) |
| Xanthophyll | Photochemical Refl. Index (515) | PRI_515_ | (R_515_-R_531_)/(R_515_+R_531_) | (A et al., 2011) |
|  | Photochemical Refl. Index (512) | PRI_m1_ | (R_512_-R_531_)/(R_512_+R_531_) | (A et al., 2011) |
|  | Photochemical Refl. Index (600) | PRI_m2_ | (R_600_-R_531_)/(R_600_+R_531_) | (Gamon et al., 1992) |
|  | Photochemical Refl. Index (670) | PRI_m3_ | (R_670_-R_531_)/(R_670_+R_531_) | (Gamon et al., 1992) |
|  | Photochemical Refl. Index (670 and 570) | PRI_m4_ | (R_570_-R_531_-R_670_)/  (R_570_+R_531_+R_670_) | (A et al., 2011) |
|  | Carotenoid/Chlorophyll Ratio Index | PRI×CI | (R_570_-R_530_)/(R_570_+R_530_)  ×((R_760_/R_700_)-1) | (Garrity et al., 2011) |
| Water | RI_1062,1393_ | RI_1062,1393_ | R_1062_/R_1393_ | (Féret et al., 2011) |
|  | ND_1062,1393_ | ND_1062,1393_ | (R_1062_-R_1393_)/  (R_1062_+R_1393_) | (Féret et al., 2011) |
|  | Water index | WI | R_900_/R_970_ | (Penuelas et al.) |
|  | Normalized Difference  Water Index | NDWI | (R_860_-R_1240_)/  (R_860_+R_1240_) | (Penuelas et al.) |
|  | Normalized Difference  Infrared Index | NDII | (R_819_-R_1600_)/  (R_819_+R_1600_) | (Hardisky et al., 1983) |
| R/G/B color | Redness Index | R | R_700_/R_670_ | (Gitelson et al., 2000) |
|  | Greenness Index | G | R_570_/R_670_ | (Calderón et al., 2013) |
|  | Blue Index | B | R_450_/R_490_ | (Calderón et al., 2013) |
|  | Blue/green index | BGI1 | R_400_/R_550_ | (Zarco-Tejada et al., 2005) |
|  | Blue/green index  Blue/red index | BGI2 | R_450_/R_550_ | (Zarco-Tejada et al., 2005) |
|  |  | BRI1 | R_400_/R_690_ | (Zarco-Tejada et al., 2012) |
|  | Blue/red index | BRI2 | R_450_/R_690_ | (Zarco-Tejada et al., 2012) |
|  | BF1 | BF1 | R_400_/R_410_ | (Zarco-Tejada et al., 2018) |
|  | BF2 | BF2 | R_400_/R_420_ | (Zarco-Tejada et al., 2018) |
|  | BF3 | BF3 | R_400_/R_430_ | (Zarco-Tejada et al., 2018) |
|  | BF4 | BF4 | R_400_/R_440_ | (Zarco-Tejada et al., 2018) |
|  | BF5 | BF5 | R_400_/R_450_ | (Zarco-Tejada et al., 2018) |
|  | Red/green index | RGI | R_690_/R_550_ | (Zarco-Tejada et al., 2005) |
|  | Ratio Analysis of Reflectance Spectra | RARS | R_746_/R_513_ | (Chappelle et al., 1992) |
|  | Lichtenthaler Index | LIC1 | (R_800_-R_680_)/(R_800_+R_680_) | (Lichtenthaler et al., 1996) |
|  |  | LIC2 | R_440_/R_690_ |  |
|  |  | LIC3 | R_440_/R_740_ |  |
| Plant stress | Health-index  (534,698,704) | HI_2013 | (R_534_-R_698_)/(R_534_+R_698_)-0.5×R_704_ | (Mahlein et al., 2013) |
|  | Health-index  (739,402,403) | HI_2014 | (R_739_-R_402_)/(R_739_+R_402_)-0.5×R_403_ | (Huang et al., 2014) |
|  | Plant Senescence Reflectance Index | PSRI | (R_680_-R_500_)/R_750_ | (Merzlyak et al., 1999) |
|  | Normalized Phaeophytinization Index | NPQI | (R_415_-R_435_)/(R_415_+R_435_) | (Penuelas et al., 1995) |
|  | Reflectance Curvature Index | CUR | $(R_{675}R_{690})/R_{683}^{2}$ | (Zarco-Tejada et al., 2000) |
|  | Simple Ratio | RR | R_695_/R_420_ | (CARTER and Gregory, 1994) |
|  |  |  | R_695_/R_670_ |  |
|  |  |  | R_695_/R_760_ |  |
|  |  |  | R_710_/R_760_ |  |

# References

A, R.H., A, N.C., B, L.S., C, F.M., and D, Z.T. (2011). Assessing structural effects on pri for stress detection in conifer forests. *Remote Sens. Environ.* 115, 2360-2375.

Anatoly, A., Gitelson, Yoav, and Zur, et al. (2002). Assessing carotenoid content in plant leaves with reflectance spectroscopy. *Photochemistry & Photobiology*.

Blackburn, G.A. (1998a). Quantifying chlorophylls and caroteniods at leaf and canopy scales: an evaluation of some hyperspectral approaches. 66, 273-285.

Blackburn, G.A. (1998b). Spectral indices for estimating photosynthetic pigment concentrations: a test using senescent tree leaves. *Int. J. Remote Sens.* 19, 657-675.

Blackburn, G.A. (1999). Relationships between spectral reflectance and pigment concentrations in stacks of deciduous broadleaves. *Remote Sens. Environ.* 70, 224-237.

Calderón, R., Navas-Cortés, J.A., Lucena, C., and Zarco-Tejada, P.J. (2013). High-resolution airborne hyperspectral and thermal imagery for early detection of verticillium wilt of olive using fluorescence, temperature and narrow-band spectral indices. *Remote Sens. Environ.* 139, 231-245.

CARTER, and Gregory, A. (1994). Ratios of leaf reflectances in narrow wavebands as indicators of plant stress. *Int. J. Remote Sens.* 15, 697-703.

Chappelle, E.W., Kim, M.S., and Iii, M.M. (1992). Ratio analysis of reflectance spectra (rars): an algorithm for the remote estimation of the concentrations of chlorophyll a, chlorophyll b, and carotenoids in soybean leaves. *Remote Sens. Environ.* 39, 239-247.

Dash, J., and Curran, P.J. (2004). Mtci: the meris terrestrial chlorophyll index. *Int. J. Remote Sens.* 25, 151-161.

Datt, B. (1998). Remote sensing of chlorophyll a, chlorophyll b, chlorophyll a+b, and total carotenoid content in eucalyptus leaves. *Remote Sens. Environ.*

Datt, B. (1999). A new reflectance index for remote sensing of chlorophyll content in higher plants: tests using eucalyptus leaves. *J. Plant Physiol.* 154, 30-36.

Féret, J., Fran Ois, C., Gitelson, A., Asner, G.P., and Barry, K.M., et al. (2011). Optimizing spectral indices and chemometric analysis of leaf chemical properties using radiative transfer modeling. *Remote Sens. Environ.* 115, 2742-2750.

Gamon, J.A., Huemmrich, K.F., Wong, C.Y.S., Ensminger, I., and Peñuelas, J. (2016). A remotely sensed pigment index reveals photosynthetic phenology in evergreen conifers. *Proceedings of the National Academy of Sciences* 113, 201606162.

Gamon, J.A., Pe Uelas, J., and Field, C.B. (1992). A narrow-waveband spectral index that tracks diurnal changes in photosynthetic efficiency. *Remote Sens. Environ.* 41, 35-44.

Gamon, J.A., and Surfus, J.S. (1999). Assessing leaf pigment content and activity with a reflectometer. *New Phytol.* 143, 105-117.

Garrity, SR, Bohrer, Maurer, and KD, et al. (2011). A comparison of multiple phenology data sources for estimating seasonal transitions in deciduous forest carbon exchange. *Agric. For. Meteorol.* 2011,151(12), 1741-1752.

Gitelson, A., and Merzlyak, M.N. (1994). Quantitative estimation of chlorophyll-a using reflectance spectra: experiments with autumn chestnut and maple leaves. *Journal of Photochemistry and Photobiology B Biology* 22, 247-252.

Gitelson, A.A., Buschmann, C., and Lichtenthaler, H.K. (1999). The chlorophyll fluorescence ratio f735/f700 as an accurate measure of the chlorophyll content in plants. *Remote Sens. Environ.* 69, 296-302.

Gitelson, A.A., Keydan, G.P., and Merzlyak, M.N. (2006). Three‐band model for noninvasive estimation of chlorophyll, carotenoids, and anthocyanin contents in higher plant leaves. *Geophys. Res. Lett.* 33, 431-433.

Gitelson, A.A., Merzlyak, M.N., and Chivkunova, O.B. (2001). Optical properties and nondestructive estimation of anthocyanin content in plant leaves. *Photochem. Photobiol.*

Gitelson, A.A., Yacobi, Y.Z., Schalles, J.F., Rundquist, D.C., and Etzion, D. (2000). Remote estimation of phytoplankton density in productive waters.

Gitelson, A.A., and Merzlyak, M.N. (1996). Signature analysis of leaf reflectance spectra: algorithm development for remote sensing of chlorophyll. *J. Plant Physiol.* 148, 494-500.

Haboudane, D., Miller, J.R., Tremblay, N., Zarco-Tejada, P.J., and Dextraze, L. (2002). Integrated narrow-band vegetation indices for prediction of crop chlorophyll content for application to precision agriculture. *Remote Sens. Environ.* 81, 416-426.

Hardisky, M., S., Klemas, V., and Smart, A.R.M. (1983). The influence of soil salinity, growth form, and leaf moisture on the spectral radiance of spartina alterniflora canopies. *Photogrammetric Eng & Remote Sens* 49, 77-84.

Huang, W., Guan, Q., Luo, J., Zhang, J., and Zhao, J., et al. (2014). New optimized spectral indices for identifying and monitoring winter wheat diseases. *Selected Topics in Applied Earth Observations and Remote Sensing, Ieee Journal of* 7, 2516-2524.

Lichtenthaler, H.K., Lang, M., Sowinska, M., Heisel, F., and Miehé, J.A. (1996). Detection of vegetation stress via a new high resolution fluorescence imaging system. *J. Plant Physiol.* 148, 599-612.

Lu, S., Lu, F., You, W., Wang, Z., and Omasa, K. (2018). A robust vegetation index for remotely assessing chlorophyll content of dorsiventral leaves across several species in different seasons. *Plant Methods* 14, 15.

Maccioni, A., Agati, G., and Mazzinghi, P. (2001). New vegetation indices for remote measurement of chlorophylls based on leaf directional reflectance spectra. *J. Photochem. Photobiol. B.* 61, 52-61.

Mahlein, A.K., Rumpf, T., Welke, P., Dehne, H.W., and Pl Mer, L., et al. (2013). Development of spectral indices for detecting and identifying plant diseases. *Remote Sens. Environ.* 128, 21-30.

Merzlyak, M.N., Gitelson, A.A., Chivkunova, O.B., and Rakitin, V.Y. (1999). Non-destructive optical detection of pigment changes during leaf senescence and fruit ripening. *Physiol. Plant.*

Penuelas, J., Baret, F., and Filella, I. (1995). Semiempirical indexes to assess carotenoids chlorophyll-a ratio from leaf spectral reflectance. *Photosynthetica* 31, 221-230.

Penuelas, J., Pinol, J., Ogaya, R., and Filella, I. Estimation of plant water concentration by the reflectance water index wi (r900/r970).

Rouse, J.W., Haas, R.W., Schell, J.A., Deering, D.W., and Harlan, J.C. (1974). Monitoring the vernal advancement and retrogradation (green wave effect) of natural vegetation. Greenbelt, md: nasa/gsfc type iii, final report.

Sims, D.A., and Gamon, J.A. (2002). Relationships between leaf pigment content and spectral reflectance across a wide range of species, leaf structures and developmental stages. *Remote Sens. Environ.* 81, 337-354.

Vogelmann, J.E., Rock, B.N., and Moss, D.M. (1993). Red edge spectral measurements from sugar maple leaves. *Int. J. Remote Sens.* 14, 1563-1575.

Zarco-Tejada, P.J., Berjón, A., López-Lozano, R., Miller, J.R., and Martín, P., et al. (2005). Assessing vineyard condition with hyperspectral indices: leaf and canopy reflectance simulation in a row-structured discontinuous canopy. *Remote Sens. Environ.* 99, 271-287.

Zarco-Tejada, P.J., Camino, C., Beck, P.S.A., Calderon, R., and Hornero, A., et al. (2018). Previsual symptoms of xylella fastidiosa infection revealed in spectral plant-trait alterations. *Nat. Plants* 4, 432-439.

Zarco-Tejada, P.J., González-Dugo, V., and Berni, J.A.J. (2012). Fluorescence, temperature and narrow-band indices acquired from a uav platform for water stress detection using a micro-hyperspectral imager and a thermal camera. *Remote Sens. Environ.* 117, 322-337.

Zarco-Tejada, P.J., Miller, J.R., Mohammed, G.H., Noland, T.L., and Sampson, P.H. (2000). Chlorophyll fluorescence effects on vegetation apparent reflectance. *Remote Sens. Environ.* 74, 596-608.
